# Association between fear of future workplace violence and burnout among pediatricians in China with psychological resilience as a moderator

**DOI:** 10.1093/joccuh/uiaf029

**Published:** 2025-05-23

**Authors:** Yuntian Shi, Fangxiang Mao, Xuan Zhang

**Affiliations:** School of Nursing and Rehabilitation, Shandong University, Jinan, Shandong Province, China; The Second Clinical Medical College, Jining Medical University, Jining, Shandong Province, China; School of Nursing and Rehabilitation, Shandong University, Jinan, Shandong Province, China; Department of Epidemiology, Erasmus Medical Center, Erasmus University Rotterdam, Rotterdam, the Netherlands; School of Nursing and Rehabilitation, Shandong University, Jinan, Shandong Province, China

**Keywords:** fear of future workplace violence, burnout, pediatrician, psychological resilience, employee resilience

## Abstract

**Objectives**: This study explored the relationship between fear of future workplace violence (FFWV) and burnout, and the moderating effect of psychological resilience on this relationship.

**Methods**: We recruited 413 pediatricians from 26 specialized and general hospitals in China’s Shandong provinces and Ningxia Hui Autonomous Region from August 2021 to April 2022. Fear of future workplace violence was measured using the Fear of Future Violence at Work Scale. Burnout was assessed using the Maslach Burnout Inventory. Psychological resilience was measured using the 10-item Connor-Davidson Resilience Scale. A multiple linear regression analysis was used to investigate the relationship between FFWV and burnout. The SPSS PROCESS macro was used to examine the moderating effect of psychological resilience on this relationship.

**Results**: About 85.7% of pediatricians experienced a medium or high level of fear. Fear of future workplace violence was significantly and positively associated with emotional exhaustion, depersonalization, and personal accomplishment (*B* = 0.23, SE = 0.39, *P* < .001; *B* = 0.06, SE = 0.13, *P* < .001; *B* = 0.17, SE = 0.03, *P* < .001, respectively, where B denotes the unstandardized regression coefficient). The interaction effects of FFWV and psychological resilience on emotional exhaustion (*B* = −0.008; 95% CI, −0.01 to −0.004) and depersonalization (*B* = −0.003; 95% CI, −0.005 to −0.001) were statistically significant. The protective effect conforms to the “protective-stabilizing” model.

**Conclusions**: Psychological resilience training may be beneficial for pediatricians in preventing high levels of emotional exhaustion and cynicism resulting from FFWV.

## Teaser

Key points:

- **What is already known on this topic:** Fear of future workplace violence (FFWV) can lead to withdrawal intentions and poor physical and mental health. However, no study has focused on FFWV among pediatricians, who may be at high risk.
- **What this study adds:** Low levels of FFWV were associated with low levels of emotional exhaustion, cynicism, and increased personal accomplishment. Psychological resilience weakened the association between FFWV, emotional exhaustion, and depersonalization.
- **How this study might affect research, practice, or policy:** Governments should develop policies that prioritize health care service safety, deter workplace violence, and impose strict penalties on aggressors, as well as provide psychological support to health care workers who have been affected by workplace violence. Psychological resilience training may be beneficial for pediatricians in preventing high levels of emotional exhaustion, and depersonalization resulting from FFWV.

## 1. Introduction

Medical practice can be a fulfilling and rewarding career; however, it also involves significant challenges and stressors. The 2023 Medscape National Physician Burnout and Depression Report^1^ surveyed over 9100 physicians in the United States across different specialties and found that 53% suffered from burnout. The World Health Organization,^2^ in the 11th Revision of the International Classification of Diseases, has classified burnout as an occupational phenomenon, defining it as a syndrome caused by “chronic workplace stress that has not been properly managed.” Maslach's 3-dimensional construct of burnout^3^ includes emotional exhaustion, depersonalization (cynicism), and reduced personal accomplishment. The impact of burnout is not limited to physicians’ well-being; it also affects the health care system, leading to reduced productivity and increased physician turnover. More importantly, it can affect patient outcomes, resulting in increased medical errors, decreased quality of patient care, longer recovery times, and decreased patient satisfaction.^4^ Therefore, physician wellness is a recommended quality measure for the delivery of health care.^5^

Pediatricians specialize exclusively in the health and development of infants, children, adolescents, and young adults.^6^ Their training is dedicated to understanding the unique physiological, psychological, and social aspects of child health, and differs significantly from the training of other medical specialists.^6^ Moreover, pediatricians often establish long-term relationships with their patients and families, providing continuous and comprehensive care throughout the various stages of a child's development.^6^ In contrast, other specialists may provide care that is more episodic or focused on specific organ systems or diseases, without the same emphasis on holistic child development. Additionally, pediatricians play a central role in coordinating care among various health care providers, ensuring that all aspects of a child's health are addressed in a cohesive manner.^6^ This coordination is particularly important in managing complex or chronic conditions that require input from multiple specialists.

However, the prevalence of burnout among pediatricians has reached high levels. In Medscape's 2023 survey, US pediatricians had the third-highest burnout rate at 59%, a significant increase from the 2019 survey,^7^ in which the burnout rate was the 17th highest at 41%. Chinese pediatricians are responsible for 10 times as many patients as their counterparts in the United States,^8^ with some tertiary children's hospitals assigning a single doctor to handle 80-100 visits per day, and work an average of 50 hours per week.^9^ Despite their heavy workload, Chinese pediatricians earn less than other senior medical service providers.^8^ These factors exacerbate burnout, which in turn may further exacerbate the shortage of pediatricians through increased turnover,^4^ creating a vicious cycle. Given the alarming prevalence and detrimental effects of burnout among pediatricians, immediate action is crucial.

Pediatricians in China suffer not only from high levels of burnout but also high levels of workplace violence.^10^ Owing to China's long-standing 1- and 2-child policies, parents and grandparents are especially concerned about their children's health. If a child's condition worsens, many families instinctively blame the doctor, regardless of the cause.^10^ Thus, the incidence of medical disputes in pediatric patients is higher than in other specialties.^10^ There were 5 cases of violence against pediatric health care workers in China in 2006, 7 in 2008, 10 in 2010, and 15 in 2013. The incidence of medical violence increases by nearly 20% each year, as reported by NetEase News (2013, as cited in Xu et al^10^). A growing body of research demonstrates that workplace violence is an important risk factor for burnout among health care workers.^11^^,^^12^ This means that the impact of workplace violence comes not only from direct experience but also from fear of future workplace violence (FFWV).^13^ Fear of future workplace violence refers to an individual's subjective feeling of risk of experiencing workplace violence, which can lead to withdrawal intentions^14^ and poor physical and mental health.^15^ Health care workers who have directly experienced or indirectly experienced it (eg, hearing or witnessing violence against health care workers) may experience fear,^16^ as some fear violence even if they have not been subjected to it during their professional lives.^13^ A recent study showed that more than 70% of nurses reported feelings of FFWV, indicating that FFWV may be more common than actual experience of workplace violence.^16^ Furthermore, Fu et al^17^ found that among nurses in China, compared with high levels of FFWV, low levels of fear were associated with lower levels of emotional exhaustion, cynicism, and personal accomplishment, whereas medium levels of fear were associated with lower emotional exhaustion and cynicism, but higher personal accomplishment. Mutlu et al^18^ found that a high fear of violence is associated with a reduced tendency to emigrate among health care workers in Turkey. Despite the high risk of workplace violence faced by pediatricians, FFWV has received little research attention in this group. To address this gap, the first aim of this study was to assess the level of FFWV among pediatricians in China and examine its association with burnout.

Notably, not all pediatricians who experience FFWV report the same levels of burnout. This suggests that other factors—particularly individual protective factors—may play a critical role in moderating this relationship. Understanding these factors is essential for developing effective interventions. The Job Demands–Resources (JD-R) model provides a useful theoretical lens for interpreting these relationships.^19^ It posits that prolonged psychological or physical effort in response to high job demands can deplete individual resources, increasing the risk of burnout and related negative outcomes. In this context, FFWV can be viewed as a situational psychological demand that contributes to burnout. However, the JD-R model also emphasizes that the availability of resources—both job-related (eg, social support, performance feedback, and motivating job characteristics) and personal (eg, optimism, self-efficacy)—can buffer these negative effects by helping individuals manage demands and maintain motivation.^20^

One such personal resource is psychological resilience, which refers to an individual’s ability to adapt positively or maintain mental well-being in the face of adversity.^21^ Although the definitions of resilience vary across the literature, some studies show that resilience should be used as a buffer against the harmful effects of stressors.^22^ Given the intensity of their work, psychological resilience may be expected to be greater among practicing physicians than among people in other careers.^23^ Physicians with higher levels of resilience may be expected to navigate the demands of their professional lives more effectively and experience lower levels of burnout.^23^ As explained by the protective effect models of psychological resilience,^24^ individuals with high levels of psychological resilience may buffer harmful effects when faced with stressful events and processes. In contrast, those with lower levels of psychological resilience are more likely to experience negative outcomes from the impact of stress. Although multiple studies have revealed the role of psychological resilience as a buffer between stress and mental health outcomes,^25^^-^^27^ it remains unclear whether psychological resilience plays a moderating role in FFWV and burnout among pediatricians.

**Figure 1:**
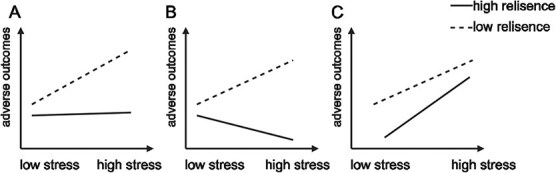
Illustrative effects of moderator variables, in interaction with stress, in relation to adverse outcomes: (A) protective-stabilizing model, (B) protective-enhancing model, (C) protective-reactive model.

Notably, Luthar^28^ has argued for the incorporation of more differentiated terms to label the protective effects of psychological resilience. There are 3 models of protective effects of psychological resilience: “protective-stabilizing,” “protective-enhancing,” and “protective-reactive.” “Protective-stabilizing” refers to when the psychological resilience confers stability in outcomes despite increasing risk (Figure 1A); “protective-enhancing” refers to situations when psychological resilience allows individuals to “engage” with stress such that their outcomes are augmented with increasing risk (Figure 1B); and “protective-reactive” refers to when psychological resilience generally weakens adverse outcomes but less so when stress levels are high than low (Figure 1C). The second aim of this study was to explore the moderating effect of psychological resilience on the relationship between FFWV and burnout among pediatricians in China. If a moderation effect existed, we would further describe the most suitable model.

For the 2 aims of this study, the following hypotheses were tested:

(a) Fear of future workplace violence is associated with burnout based on both theoretical and empirical considerations. Theoretically, the JD-R model conceptualizes FFWV as a chronic job demand that continuously depletes emotional resources, potentially leading to burnout.^19^ Empirically, existing research has shown that FFWV is associated with higher levels of burnout among nurses.^16^ However, pediatric-specific evidence is lacking, despite the high burnout rate of 59% among pediatricians in China and their heavy workloads of 80 to 100 patients per day.^1^^,^^9^ Addressing this gap is especially urgent given the ongoing pediatrician attrition crisis in the country.

(b) Psychological resilience moderates the relationship between FFWV and burnout. Although resilience has been shown to buffer the effects of stress on mental health outcomes, its role in the context of FFWV and burnout remains unexplored. Investigating this relationship is important for identifying effective protective mechanisms in a high-risk group and for addressing a critical gap in the pediatric burnout literature. We also aimed to determine whether this moderation follows a protective-stabilizing, protective-enhancing, or protective-reactive pattern. We did not establish an a priori hypothesis regarding the specific moderation effect pattern, as no previous study has explored it.

## 2. Methods

Participants were recruited from the pediatric departments of children's specialized and general hospitals in Shandong provinces and Ningxia Hui Autonomous Region, China, from August 2021 to April 2022, using an online questionnaire. The inclusion criteria were: (1) employees who have signed a labor contract with medical institutions and receive a salary from them; (2) registered physicians with a pediatric practice qualification certificate; and (3) fluent in reading Chinese. The study was approved by the Ethics Committee of the School of Nursing and Rehabilitation of Shandong University (no. 2021-R-137). With support from hospital or department management, the questionnaire was distributed to pediatricians via a Wenjuanxing link (a widely used online platform for data collection in China). Participation was voluntary. Informed consent was obtained from all participants. Prior to participation, detailed information about the study’s purpose, procedures, voluntary nature, and confidentiality assurances was provided at the beginning of the online questionnaire. Completion and submission of the questionnaire were considered to indicate informed consent.

### 2.1. Measures

#### 2.1.1. Burnout

The severity of burnout was assessed using the Maslach Burnout Inventory (MBI-GS).^3^ It consists of 22 items, each rated on a 7-point Likert scale, ranging from 0 to 6. The MBI-GS includes 3 types of burnout: emotional exhaustion, depersonalization (cynicism), and reduced personal accomplishment. For emotional exhaustion and depersonalization, scores ³27 and ³8, respectively, were classified as existence of that particular type.^29^ Those who scored £24 were considered to have reduced personal accomplishment.^29^ The Chinese version of the MBI-GS has high validity and reliability for measuring burnout among health care workers.^30^ Cronbach α values for the emotional exhaustion, depersonalization, and reduced personal accomplishment subscales were .92, .82, and .89, respectively.

#### 2.1.2. Psychological resilience

Psychological resilience was measured using the Connor-Davidson Resilience Scale (CD-RISC), a 10-item scale that measures the ability to cope with adversity.^31^ Respondents rated the items on a scale of 0 to 4. The Chinese version of the CD-RISC has demonstrated good validity and reliability.^32^ Cronbach α for the CD-RISC in this study was .98.

#### 2.1.3. F‌FVW

Fear of future workplace violence was assessed using the FFVW scale, a 12-item scale that assesses individuals' fear of violence at work in the next year.^33^ Responses to the FFVW scale are graded on a 7-level scale, ranging from 1 to 7. Total scores on the scale range from 12 to 84. A higher score indicates a higher level of fear. The scores were divided into 3 categories: low (12-36 points), medium (37-60 points), and high (61-84 points). The Chinese version of the FFVW has demonstrated good validity and reliability.^17^ Cronbach α for the FFVW in this study was .99.

#### 2.1.4. Covariates

A series of sociodemographic and work-related characteristics, including sex (male or female), age, education level (bachelor’s degree or less, master’s degree, doctoral), monthly income (<6000 yuan, ≥6000 yuan), department (pediatric internal medicine department, pediatric surgery department, pediatric health care department), and professional title (physician, doctor-in-charge, associate chief physician, chief physician), were collected.

### 2.2. Statistical analysis

Bivariate analyses, including 1-way analysis of variance, or Pearson correlation analysis, were used to explore the association between sociodemographic and work-related characteristics and burnout. A multiple linear regression analysis was used to investigate the relationship between FFWV and burnout. The SPSS PROCESS macro was used to examine the moderating effect of psychological resilience on the relationship between FFWV and burnout. The bootstrap method, with 5000 repetitions and a 95% CI, was used to test the significance of the moderating effect. A visual representation of the interaction effects was created using interActive^34^ (https://connorjmccabe.shinyapps.io/interactive/). All statistical analyses were performed using SPSS 21.0. A 2-tailed significance level of α = .05 was used.

## 3. Results

### 3.1. Participants’ characteristics

A total of 413 pediatricians from 26 hospitals completed the questionnaires. Participants had an average age of 36.90 years; 74.8% were male, 58.8% held a master’s degree, and 85.0% specialized in pediatric internal medicine. The other sociodemographic and work-related characteristics are presented in Table 1. Among the pediatricians, 56.2% had at least 1 type of burnout, 16.9% experienced a medium level of fear of violence, 68.8% experienced a high level of fear of violence, and 85.7% experienced a medium or higher level of fear (Table S1).

**Table 1:** Descriptive statistics of demographic and working characteristics and their association with emotional exhaustion, depersonalization, and personal accomplishment (*n* = 413).

**Variables**	***n* (%)**	**Emotional exhaustion**			**Depersonalization**			**Personal accomplishment**		
		**Mean ± SD**	***F/r***	***P***	**Mean ± SD**	***F/r***	***P***	**Mean ± SD**	***F/r***	***P***
Sex			0.38	.539		2.36	.125		0.86	.356
Male	104 (25.2)	19.11 ± 14.44			6.87 ± 7.22			30.64 ± 13.14		
Female	309 (74.8)	20.02 ± 12.58			5.75 ± 6.12			31.94 ± 12.03		
Age	—	—	−0.12	.018	—	−0.14	.005	—	0.17	.001
Educational level										
Bachelor or less	95 (23.0)	19.29 ± 12.70	0.30	.744	6.08 ± 6.57	0.01	.988	32.23 ± 12.33	0.364	.695
Master	243 (58.9)	19.67 ± 12.82			5.99 ± 6.16			31.18 ± 12.17		
Doctor	75 (18.1)	20.79 ± 14.34			6.09 ± 7.14			32.23 ± 12.87		
Monthly income, yuan			0.23	.634		5.64	.018		4.76	.030
<6000	96 (23.2)	20.34 ± 14.48			7.38 ± 7.61			29.22 ± 13.12		
³6000	317 (76.8)	19.61 ± 12.62			5.61 ± 5.97			32.33 ± 11.99		
Department			0.003	.960		0.11	.745		0.84	.359
Pediatric internal medicine department	351 (85.0)	19.80 ± 12.88			5.99 ± 6.35			31.38 ± 12.56		
Others^a^	62 (15.0)	19.71 ± 14.13			6.27 ± 6.88			32.94 ± 10.89		
Professional title			2.60	.052		2.31	.076		5.75	.001
Physician	129 (31.2)	19.49 ± 14.00			6.36 ± 6.84			30.03 ± 12.10		
Doctor-in-charge	186 (45.0)	21.26 ± 13.00			6.35 ± 6.40			30.67 ± 12.41		
Associate chief physician	66 (16.0)	18.69 ± 11.76			5.82 ± 6.13			33.77 ± 12.52		
Chief physician	32 (7.8)	14.72 ± 10.82			3.25 ± 4.79			38.97 ± 9.11		

a Pediatric surgery and child health care departments.

**Table 2:** Association of fear of future workplace violence (FFWV) with emotional exhaustion, depersonalization, and personal accomplishment (*n* = 413).

**Variables** ^**a**^	***β***	***B***	**SE**	***P***	***R*** ^***2***^	***F***
Model 1 (Emotional exhaustion)						
FFWV	.40	0.23 (0.18,0.28)	0.03	<.001	0.18	8.99
Model 2 (Depersonalization)						
FFWV	.19	0.06 (0.03, 0.09)	0.01	<.001	0.08	3.61
Model 3 (Personal accomplishment)						
FFWV	.30	0.17 (0.12, 0.22)	0.03	<.001	0.15	7.34

a The covariates included sex, age, educational level, monthly income, department, and professional title. *B*, unstandardized regression coefficient.

### 3.2. Bivariate analysis

As shown in Table 1, Older age was associated with lower levels of emotional exhaustion, depersonalization, and reduced personal accomplishment (*r* = −0.12, *P* = .018; *r* = −0.14, *P* = .005; *r* = 0.17, *P* = .001, respectively). A higher monthly income was associated with lower scores for depersonalization and higher scores for personal accomplishment (*F* = 5.64, *P* = .018; *F* = 4.76, *P* = .030). A higher professional title was associated with higher personal accomplishment scores (*F* = 5.75, *P* = .001).

**Figure 2:**
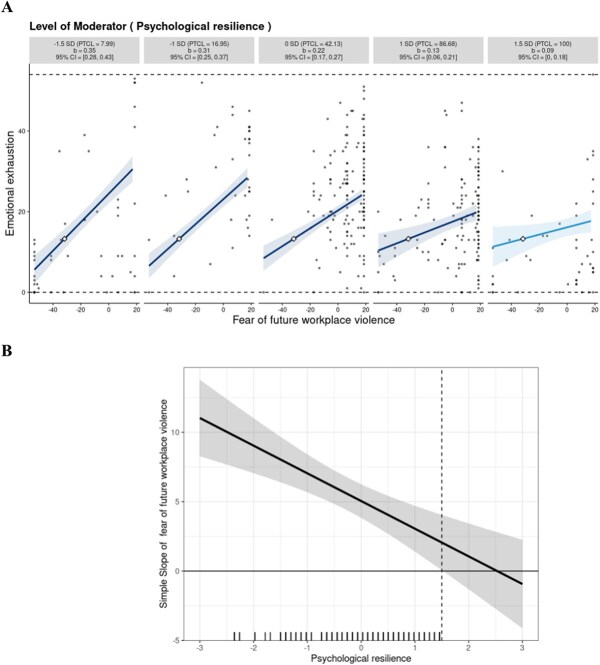
Relation between fear of future workplace violence (FFWV) and emotional exhaustion across multiples levels of psychological resilience. (A) Simple slopes for the relationship between the standardized level of FFWV(on the x-axis) and the levels of emotional exhaustion (on the y-axis) at 1.5 SD, 1 SD below the mean, mean, and 1 SD and 1.5 SD above the mean. Each graph shows the computed 95% confidence region (shaded area), observed data (gray circles), maximum and minimum values of the outcome (dashed horizontal lines), and crossover point (diamond). The x-axis represents the full range of focal predictors. (B) The marginal effect for the same interaction effect. The x-axis indicates the standardized level of the moderator and the vertical dashed lines indicate the moderator levels at which the focal variable becomes significantly associated with the dependent variable. The shaded area indicates the 95% CI. A marginal regression showing the frequency of different levels of psychological resilience was included. PTCL, percentile.

### 3.3. Multiple linear regression analysis

A regression analysis was conducted with FFWV as the independent variable and burnout as the dependent variable, while controlling for sex, age, educational level, monthly income, department, and professional title (Table 2). The results showed that FFWV was significantly and positively associated with emotional exhaustion, depersonalization, and personal accomplishment (*B* = 0.23, SE = 0.39, *P* < .001; *B* = 0.06, SE = 0.13, *P* < .001; *B* = 0.17, SE = 0.03, *P* < .001, where B denotes the unstandardized regression coefficient).

### 3.4. Moderating effect analysis

The moderating effect analysis is shown in Table 4. For emotional exhaustion, *R*^2^ increased because the interaction effect was statistically significant (Δ*R*^2^ = 0.04, *P* < .001, Model 1). The 5000-sample bootstrap procedure also demonstrated that the interaction effect was statistically significant (*B* = −0.008; 95% CI, −0.01 to −0.004). Interaction plots were created for the 5 types of psychological resilience: −1.5 SD, −1 SD, 0 SD, 1 SD, and 1.5 SD. The results showed that the regression coefficients for the 5 groups were 0.35, 0.31, 0.22, 0.13, and 0.09, respectively (Figure 2A). The regression coefficient decreased as psychological resilience scores increased, indicating that psychological resilience weakened the association between FFWV and emotional exhaustion. When an individual's psychological resilience score was at 1.5 SD, the relationship between FFWV and emotional exhaustion was not significant. The marginal-effects analysis showed that the effect of FFWV on emotional exhaustion was significant and positive when psychological resilience was 1.5 SD away from the mean or further (Figure 2B). The protective effect conforms to the “protective-stabilizing” model; that is, higher psychological resilience confers relative stability in emotional exhaustion despite increasing FFWV, compared with lower psychological resilience.

**Figure 3:**
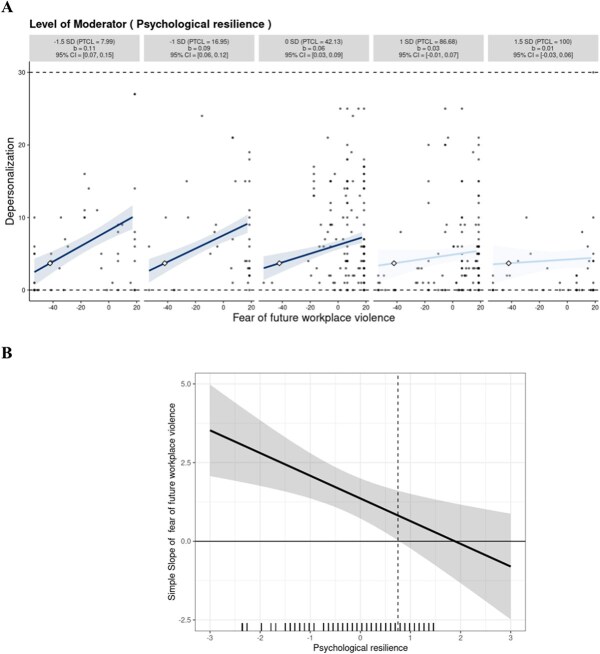
The relation between fear of future workplace violence (FFWV) and depersonalization across multiples levels of psychological resilience. (A) Simple slopes for the relationship between the standardized level of FFWV (on the x-axis) and the levels of depersonalization (on the y-axis) at 1.5 SD, 1 SD below the mean, mean, and 1 SD and 1.5 SD above the mean. Each graph shows the computed 95% confidence region (shaded area), observed data (gray circles), maximum and minimum values of the outcome (dashed horizontal lines), and crossover point (diamond). The x-axis represents the full range of focal predictors. (B) The marginal effect for the same interaction effect. The x-axis indicates the standardized level of the moderator and the vertical dashed lines indicate the moderator levels at which the focal variable becomes significantly associated with the dependent variable. The shaded area indicates the 95% CI. A marginal regression showing the frequency of different levels of psychological resilience was included. PTCL, percentile.

Similarly, for depersonalization, the 5000-sample bootstrap procedure demonstrated that the interaction effect was statistically significant (*B* = −0.003; 95% CI, −0.005 to −0.001; Table 3, Model 2). The regression coefficient decreased as psychological resilience scores increased, indicating that psychological resilience weakened the association between FFWV and depersonalization (Figure 3A). The specific protective effect conforms to “protective-reactive” effects. When an individual's psychological resilience scores were at 1 SD and 1.5 SD, the relationship between FFWV and depersonalization was nonsignificant. The marginal-effects analysis showed that FFWV on depersonalization was significant and positive when psychological resilience was 0.75 SD away from the mean or further (Figure 3B). The protective effect conforms to the “protective-stabilizing” model.

The interaction effect of FFWV and psychological resilience on personal accomplishment was not statistically significant (*B* = −0.003; 95% CI, −0.007 to 0.005; Table 3, Model 3).

## 4. Discussion

To the best of our knowledge, this is the first study to explore the association between FFWV and burnout, and the moderating effect of psychological resilience, among pediatricians. Our study found significant associations between FFWV, emotional exhaustion, depersonalization, and personal accomplishment among Chinese pediatricians. In addition, psychological resilience, as an important individual protective resource, can weaken the association between FFWV and emotional exhaustion or depersonalization.

In our study, 56.2% of participants had at least 1 type of burnout, similar to the prevalence of burnout in US pediatricians (59%), but higher than the overall prevalence of burnout among Chinese physicians (31.28%). This may be due to their low pay relative to other specialties, heavy workload, and intense patient-physician relationships.^35^ Therefore, it is important to address and prevent burnout in this population.

Older age was associated with lower emotional exhaustion, depersonalization, and reduced personal accomplishment. This phenomenon is consistent with previous findings that the prevalence of burnout is highest among early career physicians and declines with age.^36^ One possible explanation is that young health care practitioners face greater challenges in balancing their personal and professional lives, resulting in higher levels of burnout among young physicians. Unsurprisingly, our study found that higher monthly income and professional titles were associated with high personal accomplishment. As individuals progress through their careers and increase their income, they often gain access to a wider range of resources and experience an elevated sense of accomplishment.^37^ These findings indicate that young pediatricians with lower incomes and professional titles may be at a higher risk of burnout and therefore require more attention to their overall well-being.

**Table 3:** The moderation effect of psychological resilience (*n* = 413).^a^

	***β***	***B* (95%CI)**	**SE**	***P***	**Boot mean (95%CI)**	***R*** ^***2***^	**Δ*R*** ^***2***^	***F***
Model 1 (Emotional exhaustion)								
FFWV	.39	0.22 (0.17 to 0.27)	0.03	<.001	—	0.23	0.04	18.61
Psychological resilience	−.21	−0.27 (−0.39 to −0.14)	0.06	<.001	—			
FFWV × Psychological resilience	−.22	−0.008 (−0.01 to −0.004)	0.002	<.001	−0.008 (−0.01 to −0.004)			
Model 2 (Depersonalization)								
FFWV	.21	0.06 (0.03 to 0.09)	0.01	<.001	—	0.12	0.02	8.75
Psychological resilience	−.21	−0.13 (−0.19 to −0.06)	0.03	<.001	—			
FFWV × Psychological resilience	−.16	−0.003 (−0.01 to −0.001)	0.001	.003	−0.003 (−0.005 to −0.001)			
Model 3 (Personal accomplishment)								
FFWV	.14	0.08 (0.04 to 0.12)	0.02	<.001	—	0.52	0.01	4.93
Psychological resilience	.59	0.70 (0.60 to 0.79)	0.05	<.001	—			
FFWV × Psychological resilience	−.09	−0.003 (−0.006 to −0.0004)	0.002	.027	−0.003 (−0.007 to 0.005)			

Abbreviation: FFWV, fear of future workplace violence. *B*, unstandardized regression coefficient.

a Covariates include sex, age, educational level, monthly income, department, and professional title.

In our study, 85.7% of participants experienced a medium or higher level of fear, which is similar to another study among nurses in China (72.9%).^16^ In Turkey, one study examined the relationship between FFWV and health care workers’ intention to migrate, reporting a moderate mean (SD) FFWV score of 3.28 (0.71), but without providing prevalence data.^18^ Another Turkish study reported a mean score of 2.52 (0.97) among nurses.^38^ In Portugal, a study found that FFWV mediated the relationship between vicarious violence and work ability among nurses, but again only reported a mean(SD) score of 20 (10.2) (on a scale ranging from 8 to 40).^39^ Due to the use of different measurement instruments and the lack of prevalence data in studies from Turkey and Portugal, direct cross-national comparisons are limited. Nevertheless, the available evidence^38^ suggests that Chinese health care workers may experience a more pronounced FFWV compared with their counterparts in Turkey and Portugal, which may be related to the higher incidence of workplace violence in Asian countries compared with Europe.^40^ Future studies should conduct cross-cultural comparisons to understand how cultural, organizational, and systemic factors influence FFWV among health care professionals. Such studies can help identify universal and culture-specific determinants of FFWV, facilitating the development of tailored interventions.

Higher FFWV levels were associated with higher levels of emotional exhaustion. Physicians who experience emotional exhaustion describe a state of overwork and overextension.^41^ For many physicians, the inability to show empathy because of emotional exhaustion can lead to psychological distress. High workplace stress is one of the most significant causes of emotional exhaustion.^41^ Pediatricians with high fear of violence frequently experience high levels of stress, which can lead to emotional exhaustion. Similarly, higher levels of FFWV were associated with higher levels of depersonalization. Individuals experiencing burnout may become insensitive when responding to others, which is described as objectification.^41^ Fear of violence may cause physicians to feel anxious and uncomfortable when interacting with their children or caregivers. This can lead to avoidance behavior^42^ and ultimately cause emotional disengagement or apathy.

Surprisingly, a higher FFWV was associated with a higher level of personal accomplishment among pediatricians. As early as 2000, Cavanaugh et al^43^ classified stressors into challenging and hindering stress. Challenging stress, such as job overload, time pressure, and high levels of responsibility, can encourage individuals to complete work goals and realize their own abilities; however, hindrance stress, such as ambiguous role conflict, can tend to cause unnecessary resource consumption and reduce individuals’ personal sense of achievement. Pediatricians who experience high levels of fear may view their work as challenging, and working in such a vulnerable environment may lead to a greater sense of personal accomplishment. This is consistent with the professional dedication of pediatricians in overcoming challenges.^44^ Further, it is similar to the response of health care workers during the COVID-19 pandemic who showed a strong sense of accomplishment despite their high fear of infection.^45^ Research on physicians has often found that the personal accomplishment domain of burnout is only weakly correlated with outcomes.^4^ Because of this, some researchers define overall burnout as high levels of either emotional exhaustion or depersonalization.^23^ This implies that although greater fear of violence is linked to greater personal accomplishment, it does not necessarily indicate that fear of violence is beneficial.

Psychological resilience weakened the relationship between FFWV and emotional exhaustion or depersonalization among pediatricians. The specific protective effect conforms to “protective-stabilizing” effects; that is, higher psychological resilience confers relative stability in emotional exhaustion despite increased FFWV. Individuals with high psychological resilience may adopt more focused coping strategies when facing stress, such as fear of violence.^46^ They may consciously invest more effort in decision-making, approach the situation carefully and comprehensively, and avoid impulsive reactions, which can help alleviate burnout.^47^ However, research indicates that individuals with low psychological resilience may allocate more cognitive attention towards emotional input.^48^ Consequently, pediatricians with low psychological resilience may engage in repetitive recall and description of violent events that they have witnessed or experienced, leading to increased trauma-related distress and higher burnout rates. Importantly, psychological resilience is a dynamic process that can be developed.^49^ Therefore, psychological resilience training may be beneficial for pediatricians who experience high FFWV to prevent the development of high levels of emotional exhaustion and depersonalization.

Resilience training focuses on strengthening both individual and relational protective factors to enhance adaptability in adversity.^50^ At the individual level, interventions target cognitive and emotional skills, such as cultivating optimism (eg, reframing negative thoughts) and improving cognitive appraisal abilities (eg, flexible evaluation of stressors) to foster adaptive responses.^51^ Relationally, training emphasizes social skills development, including effective communication and empathy-building exercises, to strengthen interpersonal connections. These skills help individuals to access meaningful relationships that bolster resilience through mechanisms like social support, a sense of safety, and belonging.^52^^,^^53^ A systematic review and meta-analysis^54^indicated that resilience interventions for health care professionals may be more effective than control conditions in improving resilience, self-reported symptoms of depression, and stress or perceived stress post-test. However, the certainty of the evidence remains very low, highlighting the clear need for high-quality replications and more rigorous study designs.

Although our study did not directly assess the intention to migrate, existing literature indicates a significant association between workplace violence and turnover intention among health care professionals in China. For instance, Yang et al^55^ conducted a cross-sectional survey among Chinese health care workers and found that workplace violence had both direct and indirect effects on turnover intention, with perceived social support and mental health acting as mediators. Similarly, a study by Liu et al revealed that workplace violence was positively associated with turnover intention among Chinese nurses, with perceived organizational support serving as a mediator.^46^ These findings suggest that FFWV may contribute to health care workers' intentions to leave their current positions or even the profession, which could be interpreted as a form of migration within the health care sector. It is noteworthy that a recent study from Turkey found a strong association between high levels of fear of violence and a reduced tendency to emigrate among health care workers.^18^ However, another recent study from Turkey found that fear of violence is positively associated with the intention to migrate among nurses.^38^ Future research should consider including measures of migration intention to explore this potential relationship further. Additionally, psychological resilience may interact with other organizational variables. Future research could expand on our findings by examining how psychological resilience interacts with variables such as organizational commitment, job satisfaction, and psychological well-being.

This study has some limitations. First, an online self-report questionnaire may introduce the risk of recall and selection biases, potentially compromising the accuracy and objectivity of the data. In addition, social desirability bias may have influenced participants’ responses, particularly regarding sensitive topics such as fear and burnout. Second, the cross-sectional design did not permit the identification of causal relationships. Although the observed associations between fear of future workplace violence, burnout, and psychological resilience offer valuable insights, the temporal sequencing and directionality of these relationships remain unclear. Longitudinal studies are needed to examine how FFWV evolves over time and whether it contributes to subsequent changes in burnout. Third, the participants were recruited from China, hence the findings may not be generalizable to pediatricians in other countries with different health care systems, workplace cultures, or sociopolitical contexts. Future research should consider cross-national comparisons to assess the applicability of these associations in diverse global settings. Additionally, marital status was not collected in this study. Future research should consider examining the potential confounding effects of marital status on the relationship between FFWV, burnout, and psychological resilience. Lastly, the primary focus of this research was on FFWV rather than actual experiences of workplace violence. The measurement scale used assesses general fear without distinguishing between different types of violence, such as physical, verbal, or psychological. As a result, we were unable to examine whether specific types of violence are differentially associated with burnout dimensions. Future research should consider using more comprehensive tools that capture various forms of workplace violence to deepen the understanding of how different threats contribute to emotional exhaustion, depersonalization, and personal accomplishment.

To enhance health care safety and mitigate workplace violence, several policy recommendations should be considered. Firstly, establishing a nationwide early warning and reporting system for workplace violence incidents in hospitals could effectively monitor risks and enable timely interventions. Secondly, it is crucial to strengthen legal protections for health care workers, including strict enforcement of penalties for acts of violence against medical staff. Thirdly, comprehensive training programs on conflict de-escalation and communication skills should be integrated into continuing medical education. Despite these needs, current responses to workplace violence in China are predominantly managed by public security departments, with limited integration into occupational health and safety frameworks.^56^ This fragmented approach may hinder the development of more proactive and preventive strategies. Recognizing workplace violence as an occupational hazard would allow for a more systematic response—enabling routine risk assessments, targeted interventions, and support systems for affected health care workers. Internationally, "zero tolerance" policies have been adopted in countries like the United Kingdom and Australia to better protect health care staff from violence.^57^^,^^58^ These initiatives provide useful reference points for policy development.

## 5. Conclusions

This study is the first to investigate the association between FFWV and burnout among pediatricians, as well as the moderating role of psychological resilience. Our findings indicate that FFWV is highly prevalent in this population and is significantly associated with elevated levels of emotional exhaustion, depersonalization, and, unexpectedly, personal accomplishment. Psychological resilience attenuates the relationship between FFWV and both emotional exhaustion and depersonalization, demonstrating a protective-stabilizing effect. These findings highlight the need to recognize FFWV as a critical occupational issue and underscore the potential benefits of resilience-enhancing interventions for pediatricians. Strengthening individual coping resources should be accompanied by broader systemic efforts, including improved workplace safety, stronger legal protections, and the integration of violence prevention into occupational health strategies. Future studies should examine these relationships longitudinally and across diverse health care settings to inform the development of targeted and context-sensitive interventions.

## Supplementary Material

Web_Material_uiaf029

## Data Availability

The datasets used during the current study are available from the corresponding author on reasonable request.
