# Supplementary material for: Association between fear of future workplace violence and burnout among pediatricians in China with psychological resilience as a moderator
Source: J Occup Health. 2025 May 23;67(1):uiaf029. doi: 10.1093/joccuh/uiaf029 (PMC12202324; doi:10.1093/joccuh/uiaf029)
Supplement: Web_Material_uiaf029 [file web_material_uiaf029.docx]

**Supplement Table 1. The descriptive statistics of burnout and fear of future workplace violence (N=413)**

|  | Scores  Mean ± Standard deviation | Prevalence  N (%) |
| --- | --- | --- |
| Emotional exhaustion | 19.79±13.06 | 126(30.5%) |
| Depersonalization | 6.03±6.43 | 125(30.3%) |
| Personal accomplishment | 31.61±12.32 | 101(24.8%) |
| Fear of future workplace violence | 65.36±22.75 | 354(85.7%) |
